# Higher plasma aldosterone concentrations in patients with aortic diseases and hypertension: a retrospective observational study

**DOI:** 10.1186/s40001-023-01528-2

**Published:** 2023-11-26

**Authors:** Yuting Pu, Guifang Yang, Xiaogao Pan, Yang Zhou, Aifang Zhong, Ning Ding, Yingjie Su, Wen Peng, Mengping Zeng, Tuo Guo, Xiangping Chai

**Affiliations:** 1 Department of Emergency Medicine, The Second Xiangya Hospital, Central South University, 139 Renmin Road, Changsha, 410011 Hunan China; 2 Emergency Medicine and Difficult Disease Institute, The Second Xiangya Hospital, Central South University, Changsha, Hunan China

**Keywords:** Aortic diseases, Aldosterone, Hypertension, Aortic dissection

## Abstract

**Background:**

Aortic diseases remain a highly perilous macrovascular condition. The relationship between circulating aldosterone and aortic diseases is rarely explored, thus we investigated the difference in plasma aldosterone concentration (PAC) between patients with and without aortic disease in hypertensive people.

**Methods:**

We analyzed 926 patients with hypertension, ranging in age from 18 to 89 years, who had their PAC measured from the hospital's electronic database. The case group and control group were defined based on inclusion and exclusion criteria. The analysis included general information, clinical data, biochemical data, and medical imaging examination results as covariates. To further evaluate the difference in PAC between primary hypertension patients with aortic disease and those without, we used multivariate logistic regression analysis and also employed propensity score matching to minimize the influence of confounding factors.

**Results:**

In total, 394 participants were included in the analysis, with 66 individuals diagnosed with aortic diseases and 328 in the control group. The participants were predominantly male (64.5%) and over the age of 50 (68.5%), with an average PAC of 19.95 ng/dL. After controlling for confounding factors, the results showed hypertension patients with aortic disease were more likely to have high PAC levels than those without aortic disease (OR = 1.138, 95% CI [1.062 to 1.238]). Subgroup analysis revealed consistent relationship between PAC and primary hypertensive patients with aortic disease across the different stratification variables. Additionally, hypertensive patients with aortic disease still have a risk of higher PAC levels than those without aortic disease, even after propensity score matching.

**Conclusions:**

The results of this study suggest that primary hypertensive patients with aortic diseases have elevated levels of PAC, but the causal relationship between PAC and aortic disease requires further study.

**Supplementary Information:**

The online version contains supplementary material available at 10.1186/s40001-023-01528-2.

## Introduction

Aortic diseases encompass a broad range of conditions, including acute aortic syndromes (AAS) and aortic aneurysm (AA) [1]. AAS encompasses several life-threatening conditions that affect the aorta, such as acute aortic dissection (AAD) and intramural hematoma (IMH) [2]. According to the International Registry of Acute Aortic Dissection (IRAD), the initial mortality rate for AAD was 27.4%. More recent data from IRAD show that the in-hospital mortality rate for type A AAD was 22% and for type B AAD was 13% [3]. The updated 2019 Global Burden of Disease study database indicates that the number of deaths due to AA increased by 82.1% globally from 1990 to 2019 [4].

The various forms of aortic diseases share common pathways that ultimately result in increased stress on the aortic wall and/or rupture of the intima and/or media. Risk factors for this macrovascular disease include hypertension, a history of aortic or aortic valve disease, a family history of aortic disease (such as Marfan syndrome), previous cardiac surgery or trauma [1, 2]. Despite the availability of remedial treatment options, such as interventional and surgical procedures [1], after the onset of the disease, current research focuses on finding serologic indicators to disease prevention, early prompting, and therapeutic targets.

Aldosterone is a mineralocorticoid hormone produced by the adrenal cortex in response to the renin–angiotensin system and elevated serum potassium levels [5]. Aldosterone exerts its biological effects mainly through binding to mineralocorticoid receptors (MR) located in the cytoplasm or nucleus [6]. Evidence has shown that aldosterone contributes to myocardial fibrosis and hypertrophy [7, 8]. Animal studies have also demonstrated direct effects of aldosterone on the vascular system, including inducing oxidative stress, endothelial dysfunction, inflammation, fibrosis, and hypertrophic remodeling [9, 10]. Additionally, mouse models have shown that aldosterone can induce the formation and rupture of aortic aneurysms [11]. These findings suggest that aldosterone may play a role in the development and progression of aortic diseases. However, there is still a lack of human studies examining the relationship between plasma aldosterone concentration (PAC) and aortic diseases.

As such, the purpose of this study was to investigate the difference between PAC and aortic diseases in patients with hypertension, with the aim of providing an new serological research direction for the study of patients with aortic disease. This was done using a retrospective case–control design.

## Methods

### Data source

The study data were acquired non-selectively from the hospital electronic medical database at the Second Xiangya hospital, Central South University, China. The hospital's institutional review board provided the ethical approval for the study. No informed consent is required because the study was retrospective.

### Participants selection

**Fig. 1:**
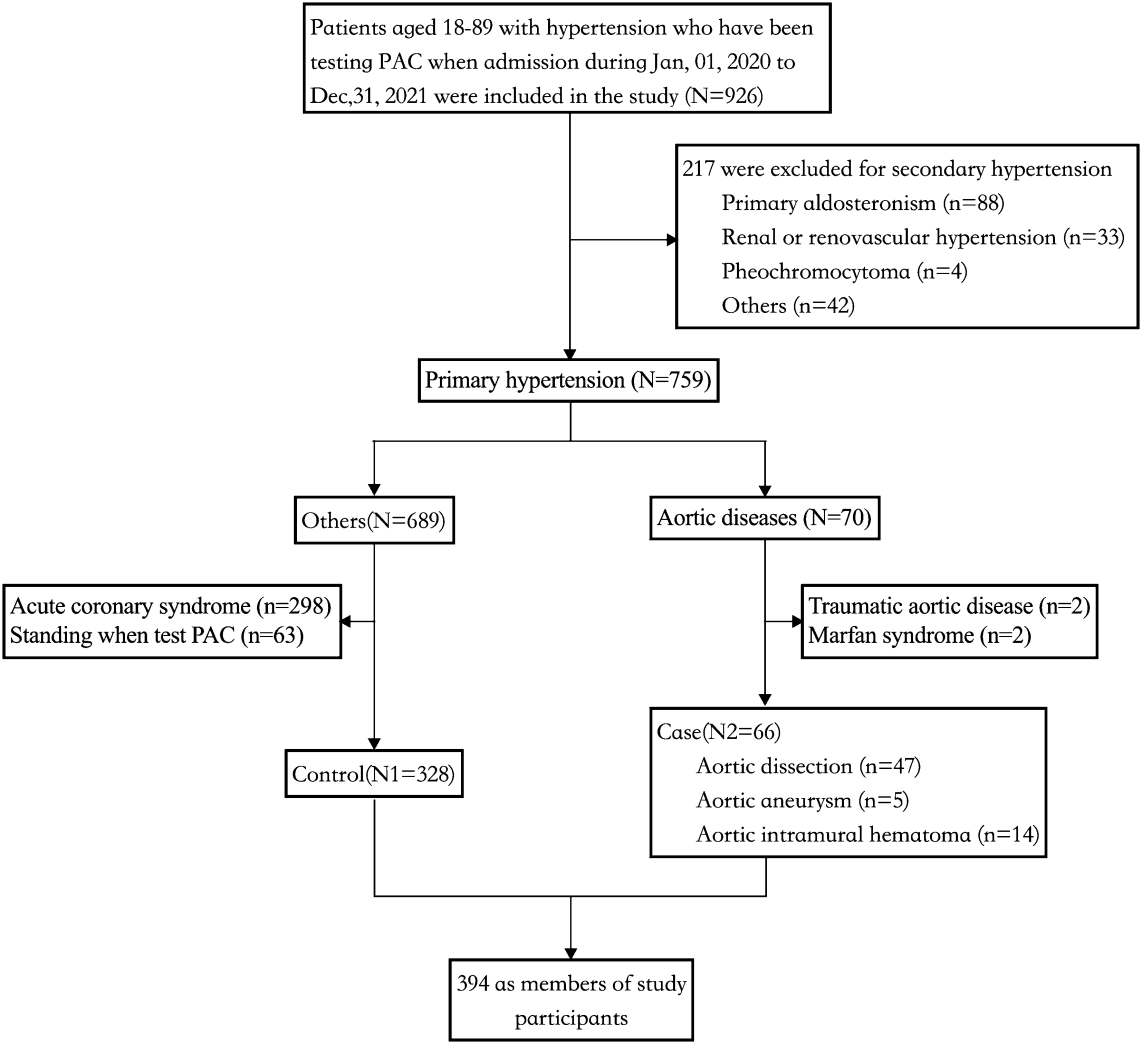
Flowchart of patient enrollment

In this analysis, 926 patients with hypertension aged 18–89 who had measured PAC during their admission to the Second Xiangya Hospital, Central South University, between January 2020 and December 2021 were included (Fig. 1). The diagnosis of aortic diseases was made primarily based on the 2014 European Society of Cardiology guidelines on the treatment and diagnosis of aortic diseases [12]. Patients who had a definite diagnosis of secondary hypertension, such as primary aldosteronism, renal or renovascular hypertension, pheochromocytoma, or could not rule out secondary hypertension, were excluded from the analysis as their data were deemed inappropriate for measuring the effect of PAC. Furthermore, patients with acute coronary syndromes, Marfan syndrome, or traumatic aortic dissection were also excluded. Additionally, patients who had their PAC measured after standing for 2 h were also excluded to eliminate any difference in PAC that may have arisen from changes in body position. Hypertensive patients with aortic disease were given to the case group, and those without aortic disease were given the contol group.

### Data collection

Data were acquired from hospital electronic medical database. Study covariates involved general data, clinical data, biochemical data, and medical imaging examination. General data included age, gender, smoking, and alcohol consumption, hospitalization, with or without intensive care, and outcome of the patient. Clinical data are divided into medical history, family medical history, medication history, and anthropometric data. Medical history include the duration of hypertension, grade of hypertension, diabetes, stroke, coronary heart disease (CHD), chronic kidney disease (CKD), adrenal lesion. Family medical history primarily refers to a family medical history of hypertension, as only 1 patient in the collected study population had a family history of aortic disease and no family history of disease affecting PAC levels. The use of angiotensin-converting enzyme inhibitor (ACEI) and/or angiotensin receptor blocker (ARB) drugs is a medication history. Anthropometric data refer to body mass index (BMI), heart rates (HR), systolic blood pressure (SBP) and diastolic blood pressure (DBP). Biochemical measurements included the levels of venous blood glucose (VBG), white blood cell (WBC), percentage of neutrophils (N%), coefficient of variation of erythrocyte distribution width (RDW-CV), serum potassium concentrations (serum K^+^), total bilirubin (TBIL), direct bilirubin (DBIL), serum creatinine (Scr), lactic acid, myoglobin, creatine kinase, creatine kinase isoenzyme MB (CK-MB), lactate dehydrogenase, N-terminal pro-brain natriuretic peptide (NT-proBNP), troponin T, triglyceride (TG), total cholesterol (TC), high-density lipoprotein cholesterol (HDL-C), low-density lipoprotein cholesterol (LDL-C), prothrombin time (PT), activated partial thromboplastin time (APTT), d-dimer (DDR), fibrin degradation products (FDP), C-reactive protein (CRP), erythrocyte sedimentation (ESR), procalcitonin (PCT), plasma renin concentrations (PRC), angiotensin II (Ang-II), PAC and adrenocorticotropic hormone. Data collected from medical imaging examination included the aortic diameter, ejection fraction, with or without aortic regurgitation.

### Definition and measurement

The classification of diseases in this study was based on the International Classification of Diseases 11, which was officially recognized by the World Health Organization on February 11, 2022. The anthropometric data analysis of the aortic disease group underwent treatment in the emergency department. The first results obtained after admission were used for all biochemical measurements and medical imaging examinations. Blood samples from patients with aortic diseases were collected within 1 h of arrival at the emergency department, while blood samples from the control group were collected within 2 h of admission. Given the unique circumstances of patients with aortic disease, all hormonal tests were performed simultaneously in the supine position.

### Missing data addressing

To ensure accurate results and minimize bias, outliers in the data were replaced with missing values. Outliers were determined as values less than the mean minus three standard deviations or more than the mean plus three standard deviations for variables with a normal distribution, and values less than the 5th percentile or greater than the 95th percentile for skewed distributions. 9 variables with a missing ratio exceeding 30%, including lactic acid, myoglobin, creatine kinase, lactate dehydrogenase, troponin T, CRP, ESR, PCT, and adrenocorticotropic hormone, were excluded from the analysis. Finally, multiple multivariable imputations were performed using the MICE package [13] to handle missing data. This resulted in the creation of 5 interpolation datasets without any missing values. A sensitivity analysis was conducted and no statistical differences were found between the interpolated dataset and the raw data. Thus, all analysis was performed based on the interpolated datasets.

### Statistical analysis

Continuous variables were expressed as mean ± normal distribution or median (25th, 75th) [skew distribution]. Categorical variables are presented as percentage. Continuous variables with normal distribution in two groups, including SBP, DBP, and N%, were compared using Student-t test. Wilcoxon rank-sum test was used for variables with skew distribution, including age, HR, DBP, BMI, duration of hypertension, VBG, WBC, RDW-CV, serum K^+^, TBIL, DBIL, Scr, CK-MB, NT-proBNP, TC, TG, HDL-C, LDL-C, APTT, DDR, FDP, PRC, Ang-II, PAC, aortic diameter and ejection fraction between two groups. Chi-squared test, Fisher test, and ANONA (one way) were applied to compare categorical variables and proportions in two groups. Additionally, PAC was applied not only as continuous variable, but also categorical variable following the median.

Multivariable logistic regression [14] was performed to investigate the risk of higher PAC levels in the case group than in the control group. The first step was to conduct a correlation analysis to assess the relationship between the independent variables. To check for multicollinearity between the covariates, variance inflation factors (VIF) were calculated in a multivariate linear model, with multicollinearity defined as VIF greater than 5. Next, univariate and multivariate regression models were performed based on the guidelines of the STROBE statement. Three different models were constructed: a crude model without adjusting for any variables, Model I adjusted for partial sociological characteristics of the population, and Model II adjusted for all variables listed in Table 1 (excluding those that were excluded due to multicollinearity or VIF > 5). The risk of high PAC levels in the case group was evaluated by increasing PAC by 1 ng/dL, 3 ng/dL, 5 ng/dL, or 7 ng/dL in the three models, and odds ratios (ORs), 95% confidence intervals (CIs), and P values were reported. At the same time, PAC was adjusted by the above three models as a categorical variable (using a median of 16.58 ng/dL). In addition, for model 2, the stability of the risk of high PAC levels was further evaluated by subgroup analysis.

**Table 1:** Clinical characteristics of study participants

	Control (*N* = 328)	Case (*N* = 66)	*P*-value
Basic information			
Age (years), median (Q1, Q3)	56.0 (44.0, 68.0)	55.0 (51.3, 60.0)	0.997
Gender (male, *n*, %)	203 (61.9%)	51 (77.3%)	0.059
Heart rates (bpm)	80.0 (71.0,91.0)	81.0 (75.0,90.0)	0.948
SBP (mmHg), mean (± SD)	151 (± 23.8)	153 (± 26.1)	0.918
DBP (mmHg), median (Q1, Q3)	90.0 (80.0, 102)	88.0 (76.5, 96.0)	0.155
BMI (kg/m^2^), median (Q1, Q3)	25.0 (22.2, 27.5)	25.6 (22.9, 28.0)	0.512
Smoking (*n*, %)	116 (35.4%)	31 (47.0%)	0.206
Alcohol consumption (*n*, %)	78 (23.8%)	11 (16.7%)	0.452
Past medical history			
DoH (years), median (Q1, Q3)	5.00 (1.00, 10.0)	5.00 (1.00, 10.0)	1
Grade 3 hypertension (*n*, %)	240 (73.2%)	40 (60.6%)	0.121
Diabetes (*n*, %)	84 (25.6%)	6 (9.1%)	0.014
Stroke (*n*, %)	54 (16.5%)	7 (10.6%)	0.487
Coronary heart disease (*n*, %)	87 (26.5%)	7 (10.6%)	0.022
Chronic kidney disease (*n*, %)	93 (28.4%)	4 (6.1%)	< 0.001
Adrenal lesion (*n*, %)	56 (17.1%)	2 (3.0%)	0.013
Family medical history			
Hypertension (*n*, %)	119 (36.3%)	9 (13.6%)	0.001
Medication history			
ACEI (*n*, %)	28 (8.5%)	14 (21.2%)	0.01
ARB (*n*, %)	113 (34.5%)	25 (37.9%)	0.868

Bpm indicates beats per minute; *SBP* systolic blood pressure, *DBP* diastolic blood pressure, *BMI* body mass index, *DoH* duration of hypertension, *ACEI* angiotensin-converting enzyme inhibitor; and *ARB* angiotensin receptor blocker

After that, propensity score matching (PSM) [15] was utilized to balance the baseline characteristics of the participants. The case group were matched 1:1 with control group participants based on their propensity score using the nearest neighbor method with a caliper of 0.02. The propensity score was calculated using a multivariate logistic regression model that took into account 13 baseline characteristics, including age, gender, BMI, duration of hypertension, presence or absence of grade 3 hypertension, diabetes, stroke, CHD, CKD, adrenal lesion, smoking, alcohol consumption and family medical history of hypertension. Finally, univariate logistic regression models were conducted for participants who underwent PSM.

A 2-tailed *P* value < 0.05 was considered significant. All the data management and analysis were operated using R version 4.9.2 for Mac.

## Results

### Clinical characteristics of participants

A total of 394 participants with hypertension were included in the study, of which 66 patients with aortic disease were included as the case group and 328 patients without aortic disease were used as the control group, based on the exclusion and inclusion criteria (Fig. 1). The baseline characteristics of these participants are shown in Table 1. The case group had a much lower percentage of post medical histories of diabetes (9.1 vs.25.6%, *P* = 0.014), CHD (10.6 vs. 26.5%, *P* = 0.022), CKD (3.0 vs.17.1%, *P* < 0.001), and adrenal lesion (3.0 vs. 17.1%, *P* = 0.013). At the same time, the case group showed significantly lower family medical history of hypertension (13.6 vs. 36.1%, *P* = 0.001), but higher using of ACEI drugs (21.2 vs. 8.5%, *P* = 0.01).

### Laboratory and imaging results of participants

As shown in Table 2, higher values of PAC (18.8 [15.5, 28.0] vs. 16.2 [11.7, 23.9] ng/dL, *P* = 0.005) was founded in the case group compared to the control group. At the same time, significantly higher values of VBG (6.70 vs. 5.17 mmol/L, *P* < 0.001), WBC (10.17 vs. 6.35 × 10^9/L, *P* < 0.001), N% (81.4 vs. 65.7, *P* < 0.001), TBIL (12.3 vs. 10.3 μmol/L, *P* = 0.006), DBIL(4.10 vs. 3.20 μmol/L, *P* < 0.001), were shown in the case group. In addition, they also had much higher values of DDR (2.32 vs. 0.300 μg/mL, *P* < 0.001) and FDP (10.3 vs. 1.92 μg/mL, *P* < 0.001). However, the case group presented lower values of LDL-C (2.34 vs. 2.71 mmol/L, *P* = 0.012), and Ang-II (11.0 vs. 12.3 ng/dL, *P* = 0.010) compared to the control group. On imaging findings, the case group had a wider aortic diameter (35 vs. 30 mm, *P* < 0.010).

**Table 2:** Laboratory and imaging findings of study participants

	Control (*N* = 328)	Case (*N* = 66)	*P*-value
Laboratory results, median (Q1, Q3)			
Venous BG (mmol/L)	5.17 (4.65, 6.04)	6.70 (5.78, 7.62)	< 0.001
WBC (× 10^9/L)	6.35 (5.18, 7.45)	10.7 (8.49, 12.6)	< 0.001
*N* (%), mean ± SD	65.7 (± 9.60)	81.4 (± 9.37)	< 0.001
RDW-CV (%)	13.0 (12.3, 13.6)	13.0 (12.4, 13.7)	0.755
Serum K^+^ (mmol/L)	3.96 (3.71, 4.27)	3.92 (3.60, 4.25)	0.875
TBIL (μmol/L)	10.3 (7.48, 13.5)	12.3 (8.65, 17.1)	0.006
DBIL (μmol/L)	3.20 (2.30, 4.20)	4.10 (3.40, 5.60)	< 0.001
Scr (μmol/L)	86.6 (66.4, 148)	93.5 (74.0, 119)	0.996
CK-MB (μg/L)	14.7 (12.3, 17.8)	13.0 (8.23, 20.6)	0.435
NT-proBNP (pg/mL)	159 (53.9, 1000)	181 (102, 660)	0.953
Triglyceride (mmol/L)	1.66 (1.20, 2.43)	1.27 (1.01, 2.06)	0.035
Total cholesterol (mmol/L)	4.39 (3.69, 5.01)	3.99 (3.62, 4.74)	0.124
HDL-C (mmol/L)	1.05 (0.868, 1.24)	1.00 (0.863, 1.17)	0.707
LDL-C (mmol/L)	2.71 (2.12, 3.31)	2.34 (1.88, 2.96)	0.012
PT (s)	12.4 (11.2, 13.2)	13.2 (12.2, 14.0)	< 0.001
APTT (s)	33.9 (28.1, 37.4)	35.0 (28.1, 38.0)	0.69
D-Dimer (μg/mL)	0.30 (0.20, 0.52)	2.32 (1.40, 4.43)	< 0.001
FDP (μg/mL)	1.92 (1.41, 2.39)	10.3 (5.19, 25.0)	< 0.001
PRC (pg/mL)	10.2 (4.72, 23.6)	7.42 (3.24, 18.7)	0.351
Ang-II (ng/dL)	12.3 (9.90, 14.4)	11.0 (8.34, 13.9)	0.010
PAC (ng/dL)	16.2 (11.7, 23.9)	18.8 (15.5, 28.0)	0.005
Imaging examination results			
Aortic diameter (mm), Median (Q1, Q3)	30.0 (28.0, 33.0)	35.0 (30.0, 42.0)	< 0.001
Aortic regurgitation (n, %)	117 (35.7%)	32 (48.5%)	0.147
Ejection fraction (%), Median (Q1, Q3)	62.0 (60.0, 64.0)	62.0 (60.0, 66.8)	0.357

BG indicates blood glucose, *WBC* white blood cell, *N* neutrophil, *RDW-CV* coefficient of variation of red blood cell distribution width; serum K^+^, serum potassium concentration, *TBIL* total bilirubin, *DBIL* direct bilirubin, *Scr* serum creatinine, *CK-MB* creatine kinase isoenzyme MB, *NT-proBNP* N-terminal pro-brain natriuretic peptide, *HDL-C* high-density lipoprotein cholesterol, *LDL-C* low-density lipoprotein cholesterol, *PT* prothrombin time, *APTT* activated partial thromboplastin time, *FDP* fibrin degradation products, *PRC* plasma renin concentration, *Ang-II* angiotensin II, *PAC* plasma aldosterone concentration

### Univariate analysis

The results of the univariate analysis showed that the case group was at risk of higher PAC (1.030 [0.983–1.005]). Additionally, the case group was positively correlated with male (2.094 [1.155–4.000]), taking ACEI drugs (2.880 [1.390–5.700]), VBG (1.697 [1.441–2.023]), WBC (1.697 [2.144–2.597]), N% (1.205 [1.157–1.261]), TBIL (1.101 [1.050–1.158]), DBIL (1.379 [1.206–1.584]), PT (1.546 [1.281–1.888]), DDR (8.717 [5.403–15.140]), FDP (2.171 [1.804–2.700]), and aortic diameter (1.697 [0.994 -2.896]). As for factors such as age, HR, BMI, smoking and alcohol consumption, duration of hypertension, stroke, RDW-CV, serum K + , CK-MB, NT-proBNP, TG, HDL-C, APTT, PRC, and ejection fraction were not associated with the aortic disease. On the other hand, patients with aortic disease are inversely associated with elevated DBP, grade 3 hypertension, diabetes, CHD, CKD, adrenal lesion, and elevated levels of Scr, TC, LDL-C, and Ang-II (Tables 3 and 4).

**Table 3:** Univariate analysis in demographic characteristics and history for prevalence of aortic diseases

	OR (95% CI)	*P*-value
Basic information		
Age (years)	1.001 (0.983–1.019)	0.908
Gender (male)	2.094 (1.155–4.000)	0.019
Heart rates (bpm)	1.000 (0.982–1.019)	0.994
Systolic blood pressure (mmHg)	1.002 (0.991–1.013)	0.676
Diastolic blood pressure (mmHg)	0.981 (0.964–0.997)	0.021
Body mass index (kg/m^2^)	1.039 (0.972–1.110)	0.249
Smoking	1.597 (0.934–2.724)	0.085
Alcohol consumption	0.641 (0.305–1.242)	0.210
Past medical history		
Duration of hypertension (years)	0.998 (0.962–1.033)	0.937
Grade 3 hypertension	0.564 (0.327–0.987)	0.042
Diabetes	0.290 (0.109–0.646)	0.006
Stroke	0.602 (0.240–1.310)	0.234
Coronary heart disease	0.334 (0.135–0.713)	0.009
Chronic kidney disease	0.163 (0.049–0.410)	< 0.001
Adrenal lesion	0.151 (0.024–0.505)	0.010
Family medical history		
Hypertension	0.277 (0.124–0.554)	< 0.001
Medication history		
ACEI	2.880 (1.390–5.770)	0.003
ARB	1.160 (0.660–1.990)	0.595

Bpm indicates beats per minute, *ACEI* angiotensin-converting enzyme inhibitor, and *ARB* angiotensin receptor blocker

**Table 4:** Univariate analysis in laboratory and imaging findings for prevalence of aortic diseases

	OR (95% CI)	*P*-value
Laboratory results		
Venous blood glucose (mmol/L)	1.697(1.441–2.023)	< 0.001
WBC (× 10^9/L)	2.144 (1.817–2.597)	< 0.001
N (%)	1.205 (1.157–1.261)	< 0.001
RDW-CV (%)	1.082 (0.873–1.318)	0.447
Serum K^+^ (mmol/L)	0.957 (0.553–1.629)	0.872
Total bilirubin (μmol/L)	1.101 (1.050–1.158)	< 0.001
Direct bilirubin (μmol/L)	1.379 (1.206–1.584)	< 0.001
Serum creatinine (μmol/L)	0.996 (0.993–0.999)	0.019
CK-MB (μg/L)	0.965 (0.917–1.016)	0.892
NT-proBNP (pg/mL)	1.000 (1.000–1.000)	0.196
Triglyceride (mmol/L)	0.821 (0.641–1.015)	0.090
Total cholesterol (mmol/L)	0.767 (0.591–0.988)	0.044
HDL-C (mmol/L)	0.629 (0.247–1.516)	0.316
LDL-C (mmol/L)	0.625 (0.458–0.842)	0.002
PT (s)	1.546 (1.281–1.888)	< 0.001
APTT (s)	1.021 (0.980–1.064)	0.314
D-Dimer (μg/mL)	8.717 (5.403–15.140)	< 0.001
FDP (μg/mL)	2.171 (1.804–2.700)	< 0.001
PRC (pg/mL)	0.995 (0.983–1.005)	0.392
Ang-II (ng/dL)	0.990 (0.982–0.997)	0.012
PAC (ng/dL)	1.030 (1.007–1.051)	0.009
Imaging examination results		
Aortic diameter (mm)	1.697 (0.994–2.896)	< 0.001
Aortic regurgitation	2.375 (1.040–5.425)	0.052
Ejection fraction (%)	1.005 (0.960–1.055)	0.84
Ejection fraction (%)	1.005 (0.960–1.055)	0.84

WBC indicates white blood cell; *N* neutrophil, RDW-CV coefficient of variation of red blood cell distribution width, serum K^+^, serum potassium concentration; *CK-MB* creatine kinase isoenzyme MB, *NT-proBNP* N-terminal pro-brain natriuretic peptide, *HDL-C* high-density lipoprotein cholesterol, *LDL-C* low-density lipoprotein cholesterol, *PT* prothrombin time, *APTT* activated partial thromboplastin time, *FDP* fibrin degradation products, *PRC* plasma renin concentration, *Ang-II* angiotensin II; PAC, plasma aldosterone concentration

### Results of multivariable logistic regression model

Correlation analysis revealed a strong positive correlation between gender and smoking, WBC, N%, Scr, NT-proBNP and CKD, SBP and DBP, TBIL and DBIL, TC and LDL-C, DDR, FDP and aortic disease. However, DBIL, Scr, HDL-C, and PT showed a high degree of collinearity (VIF > 5) and were therefore excluded from further analysis.

Three models were constructed to examine the independent effect of PAC on aortic disease after controlling for potential confounding factors (Table 5). The results showed a consistent and significant positive association between PAC and aortic disease, both in continuous and categorical forms. The odds of having aortic disease increased with each increase in PAC, regardless of the form of measurement. For example, when adjusting for confounding factors, per unit increase (1.138 [1.062–1.238], *P* < 0.001), per 3 ng/dL increase (1.475 [1.198–1.898], *P* < 0.001), per 5 ng/dL increase (1.912 [1.351–2.910], *P* < 0.001), and per 7 ng/dL increase (2.477 [1.524–4.460], *P* < 0.001) in PAC all showed significant *P* values and increased odds of aortic disease. Furthermore, when PAC was dichotomized using the median (16.58 ng/dL), the risk of aortic disease was increased by a 2.112-fold (95% CI, 1.229–13.714, *P* = 0.008) in the higher group, which was reinforced by controlling for confounding factors.

**Table 5:** Multiple logistic regression analysis for the association between PAC and aortic disease

	Unadjusted model		Model 1		Model 2	
	OR (95%CI)	*P*-value	OR (95%CI)	*P*-value	OR (95%CI)	*P*-value
PAC, ng/dL	1.030 (1.007–1.053)	0.009	1.033 (1.009–1.056)	0.006	1.138 (1.062–1.238)	< 0.001
PAC per 3 ng/dL	1.093 (1.021–1.168)	0.009	1.101 (1.027–1.179)	0.006	1.475 (1.198–1.898)	< 0.001
PAC per 5 ng/dL	1.160 (1.035–1.296)	0.009	1.174 (1.046–1.315)	0.006	1.912 (1.351–2.910)	< 0.001
PAC per 7 ng/dL	1.230 (1.050–1.437)	0.009	1.251 (1.065–1.467)	0.006	2.477 (1.524–4.460)	< 0.001
PAC median						
<16.58 ng/dL	1		1		1	
≥16.58 ng/dL	2.112 (1.229–3.714)	0.008	2.224 (1.282–3.947)	0.005	8.230 (1.728–54.074)	0.015

Model 1 was adjusted for age, gender. Model 2 was adjusted for gender, BMI, smoking, duration of hypertension, grade 3 hypertension, diabetes, stroke, CHD, CKD, adrenal lesion, family medical history, medication history of ACEI and ARB, heart rates, SBP, DBP, HR, VBG, WBC, N%, RDW-CV, K^+^, TBIL, CK-MB, NT-proBNP, TG, LDL-C, APTT, PRC, Ang-II, PAC, aortic diameter, aortic regurgitation and ejection fraction. BMI, body mass index; CHD, coronary heart disease, CKD, chronic kidney disease; ACEI, angiotensin-converting enzyme inhibitor; ARB, angiotensin receptor blocker; SBP, systolic blood pressure; DBP, diastolic blood pressure; HR, heart rates; VBG, venous blood glucose; WBC, white blood cell; N, neutrophil; RDW-CV, coefficient of variation of red blood cell distribution width; K^+^, serum potassium concentration; TBIL, total bilirubin; CK-MB, creatine kinase isoenzyme MB, NT-proBNP, N-terminal pro-brain natriuretic peptide; TG, triglyceride; LDL-C, low-density lipoprotein cholesterol; APTT, activated partial thromboplastin time; PRC, plasma renin concentration, Ang-II, angiotensin II; PAC, plasma aldosterone concentration

### Subgroup analysis

**Fig. 2:**
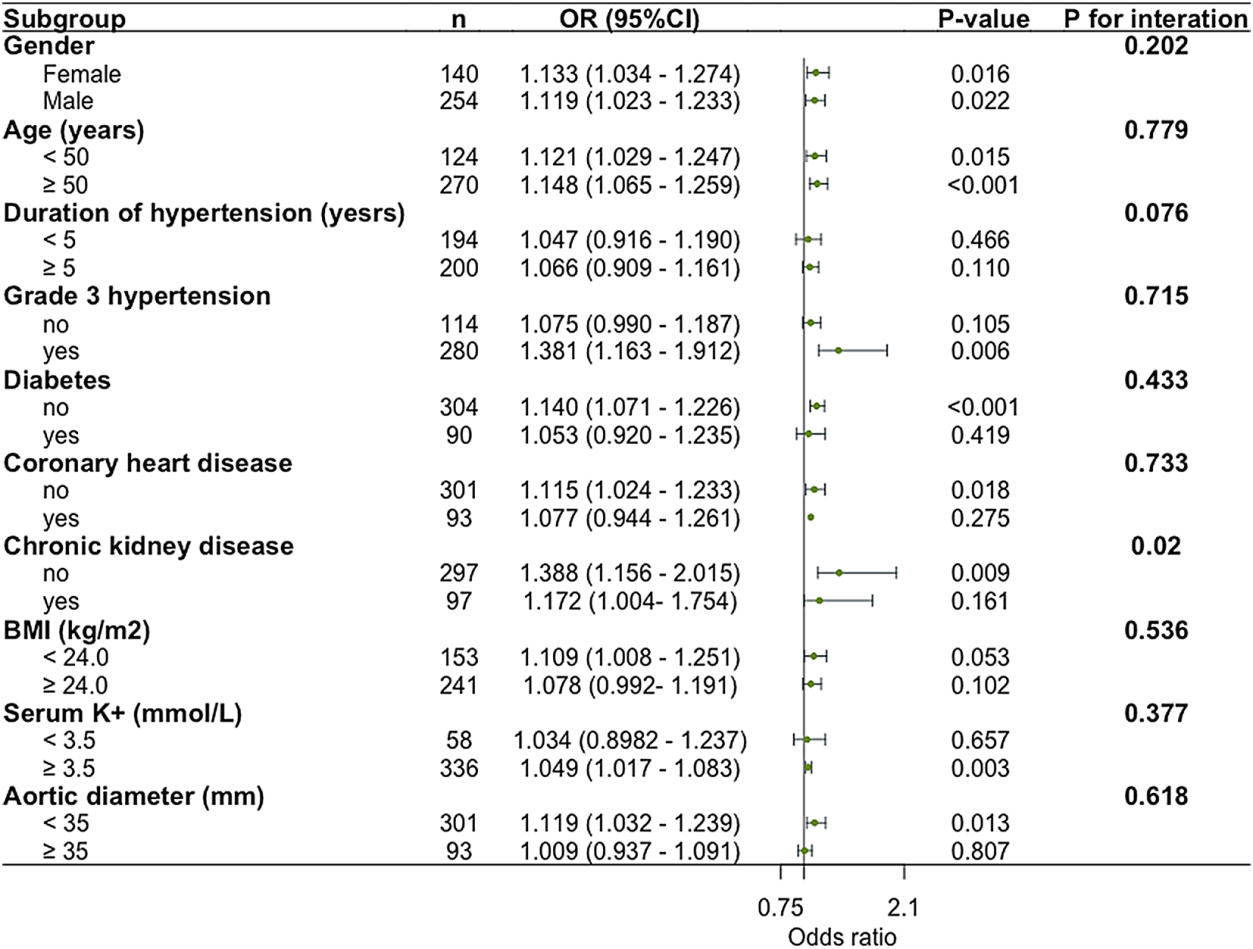
Subgroup analysis. *BMI* body mass index

Subgroup analysis was conducted based on age (< 50 and ≥ 50 years), gender, duration of hypertension (< 5 and ≥ 5 years), presence or absence of grade 3 hypertension, diabetes, CHD, CKD, BMI (< 24.0 kg/m2 and ≥ 24.0 kg/m2), serum K + (< 3.5 mmol/L and ≥ 3.5 mmol/L), and aortic diameter (< 35 mm and ≥ 35 mm). The results from the adjusted model are depicted in Fig. 2. The analysis shows that the multivariate logistic regression model established in this study is stable, and it can be considered that the relationship between PAC level and aortic disease is not affected by stratified stratification.

### Participants after PSM

In the case–control study, 80 participants were selected based on the matching of 13 baseline characteristics, including age, gender, BMI, duration of hypertension, presence or absence of grade 3 hypertension, diabetes, stroke, CHD, CKD, adrenal lesion, smoking, alcohol consumption and family medical history of hypertension. The average age of all participants was 58.34 years, with a mean PAC level of 19.10 ng/dL and 69% of the participants being male. The results of the PSM showed that the case group still had higher PAC levels (19.4 ng/dL) compared to the control group (13.8 ng/dL). And, participants with aortic disease still had significant differences from the control group in VBG, WBC, N%, DDR, FDP, PAC, and aortic diameter. Additionally, the case group had higher levels of Scr (90.9 vs. 77.0 μmol/L) compared to the control group, which was opposite before PSM (Additional file 1: Table S1).

### Univariate analysis for participants after PSM

Univariate analysis found a positive correlation between the case group and higher PAC levels (1.01 [1.01 – 1.02], P < 0.001), but no statistical relationship was observed in Ang-II levels. The case group also showed similar correlations in WBC, N%, VBG, DDR and FDP levels compared to the control group before PSM (Additional file 1: Table S1).

## Discussion

Despite the reduction in mortality rates due to advancements in diagnostic and therapeutic methods over the past two decades [16], aortic disease continues to be a serious and potentially fatal condition. Researchers are currently focused on identifying effective drug intervention targets for aortic diseases. Previous studies have indicated that aldosterone affects the cardiovascular system through various mechanisms. As a result, we conducted a case–control study to examine the relationship between PAC and aortic disease in hypertensive patients and found a positive correlation.

Aldosterone is a hormone produced by the adrenal glands and plays a role in regulating blood pressure through various mechanisms such as regulating mineralocorticoid receptors in response to angiotensin II, adrenotropic hormone, and high potassium levels [9, 17]. Aldosterone regulates blood pressure by regulating hemodynamic or non-hemodynamic changes [18–20]. Long-term exposure to elevated levels of aldosterone can lead to damage in multiple systems, including the cardiovascular system [21–23]. Hypertension is a well-established risk factor for aortic diseases [3], and thus, hypertensive patients were included in the analysis to investigate any differences in PAC in patients with and without aortic disease.

The main pathology of aortic diseases is believed to be an abnormal mesangial structure, including cystic necrosis of vascular smooth muscle and the phenotypic transformation of vascular smooth muscle cells (VSMCs)) [24, 25]. Aldosterone has been shown to act on the cardiovascular system through both the MR pathway and the MR-independent pathway [26–29]. Aldosterone binding to the cytosolic MR activates gene transcription directly or by binding to membrane-associated MR, producing factors such as cyclic adenosine 3′5-monophosphate [22] and epidermal growth factor receptor (EGFR)[30]. On the other hand, aldosterone can act on G protein-coupled estrogen receptor [31] or EGFR through non-MR or angiotensin II (1) receptors (AT1R) [32], producing reactive oxygen species [33] and mitogen-activated protein kinase [34], leading to biological activity in VSMCs via non-genetic pathways [35, 36]. Among them, the upregulation of G protein-coupled recepto-kinase (GRK)-2 plays an important role in aldosterone-mediated heart injury[37], and inhibition of GRK2 can promote post-injury intimal formation and vascular hyperplasia [38], thereby exerting a protective effect on the cardiovascular system.These multiple pathways can ultimately lead to inflammation [18], hypertrophy [39], remodeling [40], and fibrosis [41, 42] which can cause structural disruption of the intima and reduce the stiffness and compliance of the vessel wall, potentially playing a role in the development of aortic diseases.

In the study by Qing Zhu, et al., the correlation between PAC and aortic aneurysm and dissection was first examined in humans and a positive relationship was discovered [43]. Similarly, our research found that the PAC levels were higher at the onset of aortic disease in comparison to those without the disease. Primary aldosteronism has been found to be a potential cause of aortic dissection and early diagnosis and treatment of primary aldosteronism reduces the occurrence of aortic dissection [44, 45]. Previous studies have shown that the use of ARBs induces AT1R "biased" inverse agonism, thereby inhibiting aldosterone secretion [46], so we included the situation of ARB use in hypertensive patients. However, no statistically significant differences were found between the case and control groups in the use of ARB drugs. At the same time, in the multivariate logistic regression model that included ARB drug use as a confounding factor, there was still a positive correlation between PAC and aortic disease.

There are some limitations in this study. First, the study was a retrospective and observational study that could not definitively establish a causal relationship between PAC levels and aortic disease in hypertensive patients. Therefore, follow-up cohort studies are needed for further clarification. Secondly, the PAC measurement data of multiple time nodes cannot be obtained in this study, which makes it difficult to further refine the relationship between PAC and aortic disease. In addition, the populations included in the studies may have been biased in selection and were not representative of the overall samples. Some severe patients may die before they arrive at the hospital or before PAC levels are measured. This study cannot assess the PAC level in this group of patients.

Our results suggest that PAC is significantly elevated in hypertensive patients with aortic disease and highlight a positive correlation between these patients and PAC levels. However, further research is needed to confirm the causal relationship between aldosterone levels and aortic disease, as well as to explore the underlying mechanisms in aortic disease. However, our study provides the basis for future studies of aldosterone in aortic disease.

## Conclusion

This study highlights the significance of aldosterone levels in hypertensive patients with aortic disease. The results showed that patients with aortic disease had higher PAC levels at admission compared to those without aortic disease, independent of plasma renin and Ang-II levels. This finding sheds light on a new direction for research into the serology of aortic diseases.

### Supplementary Information

**Additional file 1: Table S1.** Clinical characteristics of study participants after propensity score matching (PSM) and univariate analysis.

## Data Availability

Datasets used and/or analyzed in the present study can be availed from the corresponding author on reasonable request.

## Abbreviations

AAS: Acute aortic syndromes
AA: Aortic aneurysm
AAD: Acute aortic dissection
IMH: Intramural hematoma
IRAD: International Registry of Acute Aortic Dissection
MR: Mineralocorticoid receptor
PAC: Plasma aldosterone concentration
CHD: Coronary heart disease
CKD: Chronic kidney disease
ACEI: Angiotensin-converting enzyme inhibitor
ARB: Angiotensin receptor blocker
BMI: Body mass index
HR: Heart rates
SBP: Systolic blood pressure
DBP: Diastolic blood pressure
VBG: Venous blood glucose
WBC: White blood cell
N%: Percentage of neutrophils
RDW-CV: Coefficient of variation of erythrocyte distribution width
serum K^+^: Serum potassium concentrations
TBIL: Total bilirubin
DBIL: Direct bilirubin
Scr: Serum creatinine
CK-MB: Creatine kinase isoenzyme MB
NT-proBNP: N-terminal pro-brain natriuretic peptide
TG: Triglyceride
TC: Total cholesterol
HDL-C: High-density lipoprotein cholesterol
LDL-C: Low-density lipoprotein cholesterol
PT: Prothrombin time
APTT: Activated partial thromboplastin time
DDR: D-dimer
FDP: Fibrin degradation product
CRP: C-reactive protein
ESR: Erythrocyte sedimentation
PCT: Procalcitonin
PRC: Plasma renin concentrations
Ang-II: Angiotensin II
VIF: Variance inflation factors
OR: Odds ratio
CI: Confidence interval
PSM: Propensity score matching
VSMC: Vascular smooth muscle cell
EGFR: Epidermal growth factor receptor
AT1R: Angiotensin II (1) receptor
GRK: G protein-coupled recepto-kinase
