# Supplementary material for: Higher plasma aldosterone concentrations in patients with aortic diseases and hypertension: a retrospective observational study
Source: Eur J Med Res. 2023 Nov 26;28:541. doi: 10.1186/s40001-023-01528-2 (PMC10676595; doi:10.1186/s40001-023-01528-2)
Supplement: Supplementary file 1 — Additional file 1: Table S1. Clinical characteristics of study participants after propensity score matching (PSM) and univariate analysis. [file 40001_2023_1528_MOESM1_ESM.docx]

**Additional file 1**

**Table S1. Clinical Characteristics of Study Participants after Propensity Score Matching (PSM) and Univariate Analysis.**

|  | **After matching (1:1)** | | | **Univariate analysis** | |
| --- | --- | --- | --- | --- | --- |
| **Variables,**  Median (Q1, Q3)/ Mean (±SD) | **Control (N=40)** | **Case (N=40)** | ***P*-value** | **OR (95% CI)** | ***P*-value** |
| **Age** (years) | 65.0 (48.8, 72.0) | 55.5 (52.8, 62.3) | 0.444 | 0.98 (0.95-1.02) | 0.313 |
| **Gender** (male, n, %) | 27 (67.5%) | 28 (70.0%) | 0.971 | 1.12 (0.43-2.92) | 0.809 |
| **Heart rates** (bpm) | 77.6 (±12.2) | 79.1 (±14.3) | 0.88 | 1.01 (0.98-1.04) | 0.61 |
| **SBP** (mmHg) | 150 (±23.4) | 156 (±25.4) | 0.479 | 1.01 (0.99-1.03) | 0.226 |
| **DBP** (mmHg) | 88.8 (±15.6) | 87.9 (±14.1) | 0.96 | 1 (0.97-1.03) | 0.773 |
| **BMI** (kg/m^2^) | 25.7 (22.0, 27.5) | 26.4 (22.9, 28.4) | 0.584 | 1.05 (0.94-1.18) | 0.361 |
| **Smoking**(n, %) | 13 (32.5%) | 16 (40.0%) | 0.784 | 1.38 (0.56-3.5) | 0.486 |
| **Alcohol consumption** (n, %) | 7 (17.5%) | 7 (17.5%) | 1 | 1 (0.31-3.23) | >0.999 |
| **DoH** (years) | 5.00 (1.00, 14.8) | 4.00 (1.00, 10.0) | 0.87 | 0.98 (0.93-1.03) | 0.39 |
| **Grade 3 hypertension** (n, %) | 24 (60.0%) | 24 (60.0%) | 1 | 1 (0.41-2.46) | >0.999 |
| **Diabetes** (n, %) | 5 (12.5%) | 6 (15.0%) | 0.949 | 1.24 (0.34-4.65) | 0.746 |
| **Stroke** (n, %) | 8 (20.0%) | 5 (12.5%) | 0.661 | 0.57 (0.16-1.89) | 0.367 |
| **Coronary heart disease** (n, %) | 5 (12.5%) | 6 (15.0%) | 0.949 | 1.24 (0.34-4.65) | 0.746 |
| **Chronic kidney disease** (n, %) | 1 (2.5%) | 4 (10.0%) | 0.383 | 4.33 (0.61-86.91) | 0.199 |
| **Adrenal lesion** (n, %) | 2 (5.0%) | 2 (5.0%) | 1 | 1 (0.12-8.68) | >0.999 |
| **Family history of**  **hypertension** (n, %) | 11 (27.5%) | 7 (17.5%) | 0.564 | 0.56 (0.18-1.61) | 0.287 |
| **Medication of ACEI** | 2 (5.0%) | 7 (17.5%) | 0.209 | 4.03 (0.9-28.33) | 0.096 |
| **Medication of ARB** | 14 (35.0%) | 17 (42.5%) | 0.789 | 1.37 (0.56-3.42) | 0.492 |
| **Venous BG** (mmol/L | 5.15 (4.73, 5.74) | 6.60 (5.68, 7.65) | <0.001 | 2.41 (1.54-4.18) | <0.001 |
| **WBC (×10^9/L)** | 6.42 (5.40, 7.24) | 10.2 (8.29, 12.0) | <0.001 | 2.01 (1.53-2.84) | <0.001 |
| **N (%)** | 65.4 (±10.4) | 79.8 (±10.6) | <0.001 | 1.13 (1.08-1.2) | <0.001 |
| **RDW-CV** (%) | 13.1 (12.4, 13.8) | 12.9 (12.4, 13.8) | 1 | 1.05 (0.77-1.46) | 0.757 |
| **Serum K^+^** (mmol/L) | 3.87 (±0.412) | 4.03 (±0.539) | 0.321 | 2.07 (0.82-5.58) | 0.135 |
| **TBIL** (μmol/L) | 11.6 (8.35, 14.0) | 11.7 (8.55, 16.6) | 0.864 | 1.04 (0.96-1.14) | 0.365 |
| **DBIL** (μmol/L) | 3.45 (2.88, 4.95) | 3.70 (3.25, 5.15) | 0.422 | 1.25 (0.98-1.67) | 0.095 |
| **Scr** (μmol/L) | 77.0 (62.8, 99.6) | 90.9 (73.5, 111) | 0.172 | 1.02 (1-1.03) | 0.043 |
| **CK-MB** (μg/L) | 16.4 (13.3, 19.9) | 12.9 (8.88, 18.6) | 0.064 | 0.97 (0.91-1.02) | 0.229 |
| **NT-proBNP** (pg/mL) | 115 (48.6, 318) | 179 (108, 414) | 0.401 | 1 (1-1) | 0.422 |
| **Triglyceride** (mmol/L) | 1.74 (0.910, 2.88) | 1.37 (1.02, 2.39) | 0.923 | 0.9 (0.66-1.21) | 0.474 |
| **Total cholesterol** (mmol/L) | 4.06 (3.59, 5.15) | 4.05 (3.72, 4.84) | 0.964 | 0.86 (0.57-1.28) | 0.469 |
| **HDL-C** (mmol/L) | 1.11 (0.858, 1.29) | 0.975 (0.860, 1.25) | 0.875 | 0.58 (0.14-2.22) | 0.43 |
| **LDL-C** (mmol/L) | 2.44 (1.92, 3.32) | 2.37 (1.81, 3.04) | 0.711 | 0.73 (0.45-1.14) | 0.177 |
| **PT (S)** | 12.9 (12.1, 13.3) | 13.0 (12.5, 13.9) | 0.264 | 1.42 (1.03-2.03) | 0.043 |
| **APTT** (s) | 34.0 (±5.12) | 33.7 (±6.06) | 0.965 | 0.99 (0.91-1.07) | 0.787 |
| **D-Dimer** (μg/mL) | 0.33 (0.21, 0.52) | 2.13 (1.29, 3.11) | <0.001 | 35.67 (8.59-273) | <0.001 |
| **FDP** (μg/mL) | 1.93 (1.59, 2.23) | 8.52 (5.12, 21.4) | <0.001 | 3.1 (1.92-6) | <0.001 |
| **PRC** (pg/mL) | 7.85 (4.49, 16.0) | 6.37 (2.97, 15.2) | 0.528 | 1 (0.98-1.02) | 0.861 |
| **Ang-II** (ng/dL) | 105 (93.9, 134) | 110 (81.6, 135) | 0.769 | 0.99 (0.98-1) | 0.137 |
| **PAC** (ng/dL) | 13.8 (10.1, 18.8) | 19.4 (15.4, 28.5) | <0.001 | 1.01 (1.01-1.02) | <0.001 |
| **Aortic diameter** (mm), | 31.5 (28.0, 33.0) | 33.5 (29.0, 39.8) | 0.032 | 1.15 (1.06-1.28) | 0.005 |
| **Aortic regurgitation** (n, %) | 11 (27.5%) | 18 (45.0%) | 0.266 | 2.16 (0.86-5.61) | 0.106 |
| **Ejection fraction** (%) | 62.0 (60.0, 64.0) | 62.0 (58.8, 66.0) | 0.685 | 0.99 (0.92-1.06) | 0.703 |
|  |  |  |  |  |  |

Matching age, gender, body mass index, duration of hypertension and with or without grade 3 hypertension, diabetes, stroke, coronary heart disease, chronic kidney disease and adrenal lesion, cigarette consumption, alcohol consumption and family medical history of hypertension by Propensity Score Matching (PSM). Bpm indicates beats per minute; ACEI, angiotensin-converting enzyme inhibitor; ARB, angiotensin receptor blocker; SBP, systolic blood pressure; DBP, diastolic blood pressure; DoH, duration of hypertension; VBG, venous blood glucose; WBC, white blood cell; N, neutrophil; RDW-CV, coefficient of variation of red blood cell distribution width; serum K^+^, serum potassium concentration; TBIL, total bilirubin; DBIL, direct bilirubin; Scr, serum creatinine; CK-MB, creatine kinase isoenzyme MB, NT-proBNP, N-terminal pro brain natriuretic peptide; HDL-C, high-density lipoprotein cholesterol; LDL-C, low-density lipoprotein cholesterol; PT, prothrombin time; APTT, activated partial thromboplastin time; FDP, fibrin degradation products; PRC, plasma renin concentration, Ang-II, angiotensin II; PAC, plasma aldosterone concentration.
